# Lower Preoperative Skeletal Muscle Index in Patients with Pathological T4 Colorectal Cancer: An Exploratory Retrospective Cohort Study

**DOI:** 10.3390/medsci14030356

**Published:** 2026-06-29

**Authors:** Botond-István Kiss, Árpád Török, Daniela Tatiana Sala, Renáta Moriczi, Szabolcs-Attila Gábor, Gabriel-Mircea Muresan, Tivadar Bara, Márton-István Dénes, Szilárd-Leó Kiss, Szilárd-Leó Kiss, Orsolya Kiss-Toth, Radu-Mircea Neagoe

**Affiliations:** 1 Doctoral School of Medicine and Pharmacy, “George Emil Palade” University of Medicine, Pharmacy, Science and Technology of Târgu Mureș, 540139 Târgu Mureș, Romania; drkissbotondi@gmail.com (B.-I.K.);; 2 2nd Clinic of Surgery, Târgu Mureș, County Emergency Clinical Hospital, 540136 Târgu Mureș, Romania; 3 2nd Department of Surgery, “George Emil Palade” University of Medicine, Pharmacy, Science and Technology of Târgu Mureș, 540139 Târgu Mureș, Romania; 4 Department of Anatomy and Embryology, “George Emil Palade” University of Medicine, Pharmacy, Science and Technology of Târgu Mureș, 540139 Târgu Mureș, Romania; 5 1st Department of Gynecology and Obstetrics, “George Emil Palade” University of Medicine, Pharmacy, Science and Technology of Târgu Mureș, 540139 Târgu Mureș, Romania; 6 1st Clinic of Gynecology and Obstetrics, County Emergency Clinical Hospital, 540136 Târgu Mureș, Romania; 7 1st Clinic of Neonatology, County Emergency Clinical Hospital, 540136 Târgu Mureș, Romania

**Keywords:** skeletal muscle index, colorectal cancer, locally advanced tumor, muscle depletion, T4 colorectal cancer

## Abstract

Background/Objectives: Low skeletal muscle index (SMI) has been linked to adverse outcomes in colorectal cancer, but its association with local pathological tumor extent is less clear. This study examined whether preoperative CT-derived SMI was associated with pathological T4 disease in patients undergoing colorectal cancer resection. Methods: This retrospective single-center observational study included 147 consecutive adults who underwent colorectal resection for histologically confirmed adenocarcinoma between January 2022 and November 2025 and had suitable preoperative abdominal or abdomino-pelvic CT imaging within 90 days before surgery. Skeletal muscle area was measured on a single axial CT image at the L3 level, and SMI was calculated as muscle area/height^2^. Patients were classified as having pathological T1–3 or T4 disease. Logistic regression assessed the association between SMI, expressed per 5 cm^2^/m^2^ increase, and pathological T4 stage. Results: Patients with pathological T4 tumors had lower SMI than those with T1–3 disease (37.22 vs. 42.85 cm^2^/m^2^, *p* = 0.016). Higher SMI was associated with lower odds of T4 disease in univariable analysis (OR 0.79, 95% CI 0.66–0.94; *p* = 0.008) and after adjustment for age and sex (OR 0.77, 95% CI 0.63–0.94; *p* = 0.009). Conclusions: Lower preoperative SMI was associated with pathological T4 colorectal cancer in this cohort. Because of the retrospective observational design, causality cannot be inferred. The association should be interpreted as hypothesis-generating and may reflect reverse causality, shared inflammatory–nutritional pathways, or residual confounding.

## 1. Introduction

Colorectal cancer (CRC) remains a major public health challenge worldwide, with substantial incidence, mortality, and treatment-related burden, particularly in developed countries [1,2]. In parallel with tumor-related factors, host-related characteristics such as nutritional status, systemic inflammation, and body composition have emerged as important determinants of oncologic and surgical outcomes [3,4,5].

Reduced skeletal muscle mass and impaired muscle quality, both recognized features of cancer-related sarcopenia, have been associated with increased postoperative morbidity, reduced tolerance to oncologic treatment, and shorter survival in patients with malignancy [6,7,8]. Although it is not routinely screened for in daily oncologic practice, sarcopenia may reflect a state of reduced physiologic reserve and increased vulnerability in patients with colorectal cancer [9,10,11].

The pathophysiology of muscle depletion in cancer is multifactorial and may include reduced protein intake, tumor-related catabolism, chronic low-grade systemic inflammation, metabolic dysregulation, and progressive nutritional deterioration [12,13,14]. Because skeletal muscle also serves as the body’s largest protein reservoir, its depletion may compromise immune function, tissue repair, and the capacity to respond adequately to surgical and oncologic stress [15,16,17].

Locally advanced colorectal tumors may be associated with a greater systemic metabolic burden, potentially contributing to malnutrition, cachexia, and skeletal muscle wasting [13,14,18]. From a biological perspective, this raises the possibility that advanced local tumor stage may be linked to reduced muscle quantity and poorer muscle quality, even before treatment is initiated [19,20,21].

Computed tomography is widely regarded as the reference imaging method for body composition assessment in oncologic patients, allowing objective evaluation of skeletal muscle quantity and, to a certain extent, muscle quality [11,22,23]. Parameters derived from routine preoperative CT imaging may therefore provide clinically relevant information on host nutritional and physiologic reserve. Among these parameters, skeletal muscle index (SMI) is a standardized and reproducible marker of skeletal muscle quantity, widely used as a reliable imaging surrogate of reduced muscle reserve and cancer-related muscle wasting in oncologic patients [23,24,25].

The aim of the present study was to investigate the association between preoperative skeletal muscle depletion, assessed primarily by SMI, and local tumor advancement in patients undergoing surgery for colorectal cancer. We also explored the relationship between preoperative muscle status and other clinicopathological disease characteristics.

## 2. Materials and Methods

### 2.1. Study Design and Patient Selection

We conducted a retrospective single-center observational study of consecutive adult patients who underwent colorectal resection for histopathologically confirmed colorectal adenocarcinoma between January 2022 and November 2025. Only resection cases were considered eligible, regardless of whether reconstruction was performed with primary anastomosis or stoma formation. The study was conducted and reported in accordance with the Strengthening the Reporting of Observational Studies in Epidemiology (STROBE) statement.

To be included, patients had to meet all of the following criteria: (1) age ≥ 18 years; (2) histopathologically confirmed colorectal adenocarcinoma; (3) colorectal resection performed during the study period; (4) availability of a preoperative abdominal or abdomino-pelvic CT scan obtained within 90 days before surgery; (5) CT image quality suitable for body composition analysis at the L3 level; and (6) complete postoperative histopathological staging data.

Patients were excluded if they lacked an appropriate preoperative CT examination within the predefined 90-day interval, if CT quality did not allow reliable skeletal muscle segmentation, if postoperative pathological staging was incomplete, or if the surgical procedure did not include colorectal resection.

**Figure 1:**
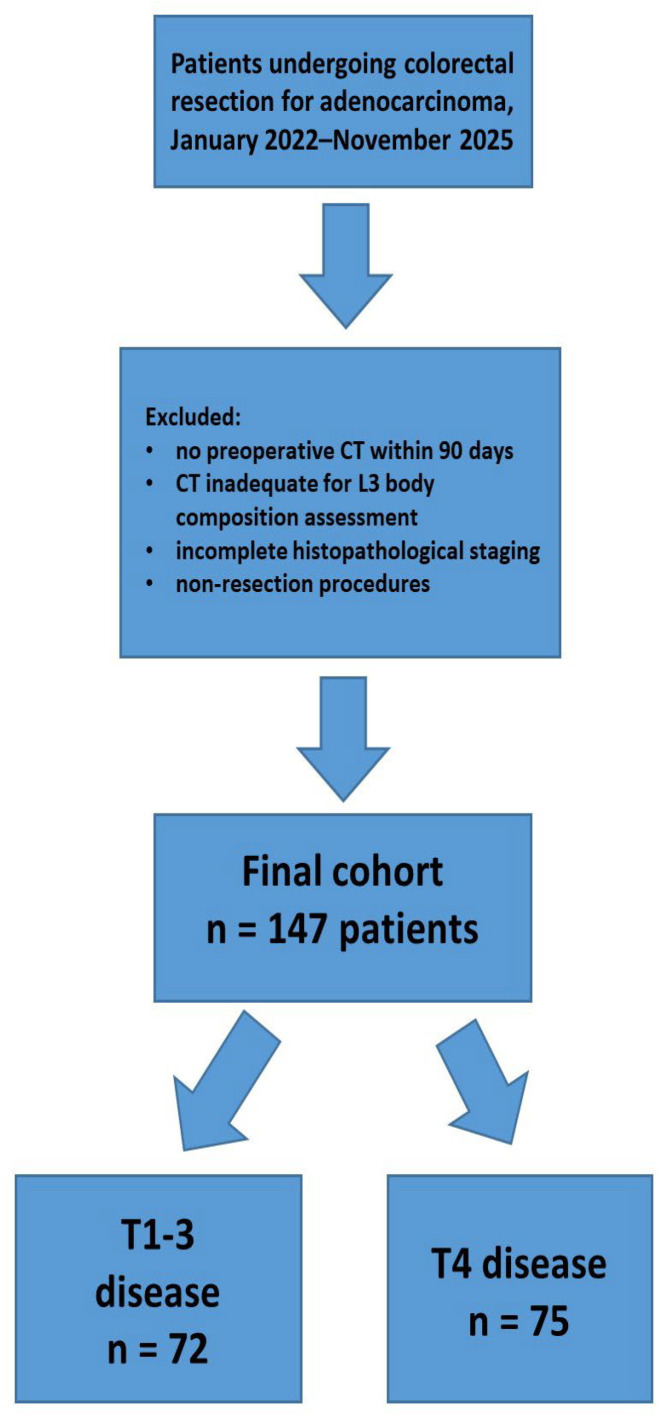
Flowchart of patient selection and final classification of the study cohort into T1–3 and T4 groups according to pathological tumor stage.

After application of the eligibility criteria, the final analytic cohort comprised 147 patients. For the primary analysis, patients were classified according to local pathological tumor stage as T1–3 or T4 disease (Figure 1).

The primary endpoint of the study was the presence of pathological T4 disease. The primary analysis was designed to evaluate whether lower preoperative skeletal muscle mass, modeled as SMI5, was associated with locally advanced tumor stage.

### 2.2. Clinical, Perioperative, and Pathological Data Collection

Clinical, perioperative, and pathological data were retrospectively retrieved from the institutional medical database (H3 Healthcare Concept System) and from histopathological reports. The collected variables included age, sex, body weight, height, body mass index (BMI), tumor localization, emergency presentation, and pathological nodal status. Tumor localization was classified as right colon, left colon, or rectum.

Pathological staging, including local tumor extent and nodal status, was established postoperatively by the attending pathologist according to internationally accepted TNM classification criteria [26]. For the primary analysis, patients were grouped according to local tumor invasion as T1–3 versus T4 disease.

### 2.3. CT-Based Body Composition Analysis

Preoperative native abdominal or abdomino-pelvic CT scans obtained within 90 days before surgery were used for body composition analysis. Images were acquired at different institutions and on different scanners, reflecting real-world clinical practice; detailed scanner-vendor information was not consistently available. Axial images with slice thickness ranging from 3 to 5 mm were reviewed. Body composition assessment was performed on a single axial image at the L3 level, selected where the transverse processes were visible bilaterally. A predefined attenuation threshold of −30 to +150 Hounsfield units (HU) was then applied to generate a skeletal muscle mask [23,27]. The entire skeletal muscle compartment at the L3 level was segmented, including the paraspinal muscles, multifidus/erector spinae, quadratus lumborum, iliopsoas, rectus abdominis, transversus abdominis, and the internal and external oblique muscles. Total cross-sectional skeletal muscle area at the L3 level was measured in cm^2^, and the skeletal muscle index (SMI) was calculated as SMA/height^2^ (cm^2^/m^2^). For an additional clinically oriented categorical analysis, CT-defined low SMI was defined using established sex- and BMI-specific cutoffs: SMI < 41 cm^2^/m^2^ in women, <43 cm^2^/m^2^ in men with BMI < 25 kg/m^2^, and <53 cm^2^/m^2^ in men with BMI ≥ 25 kg/m^2^ [24]. Because muscle strength and physical performance were not available, this variable was interpreted as CT-defined low muscle mass rather than formal sarcopenia. In addition, mean skeletal muscle radiodensity was recorded in Hounsfield units (HU).

**Figure 2:**
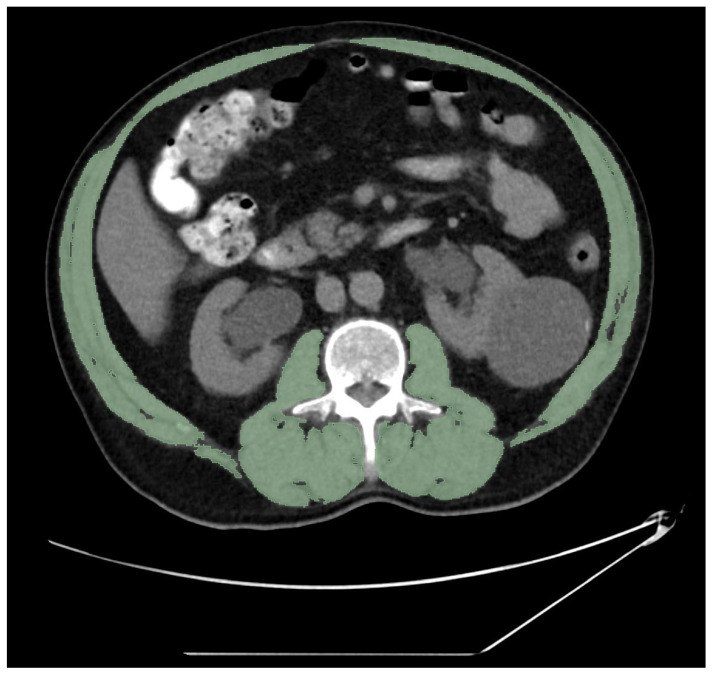
Representative axial CT image at the L3 level showing skeletal muscle area segmentation. The highlighted region represents the segmented skeletal muscle compartment used for SMI calculation. Segmentation was performed using a −30 to +150 HU threshold, followed by manual correction when necessary.

Segmentation was performed in 3D Slicer (version 5.8.1) using threshold-assisted manual segmentation. After application of the predefined attenuation threshold, the automatically highlighted muscle compartment was manually reviewed and corrected slice-by-slice when necessary to exclude non-muscular structures and ensure anatomical accuracy. A representative example of skeletal muscle area segmentation at the L3 level is shown in Figure 2.

Muscle segmentation was done by two trained observers under radiologist supervision, who were blinded to pathological findings and clinical outcomes [28]. Although segmentation was performed according to a standardized threshold-assisted protocol by trained observers under radiologist supervision, formal intraobserver and interobserver reliability testing was not performed.

### 2.4. Statistical Analysis

Statistical analysis was performed using IBM SPSS Statistics for Windows, version 25.0 (IBM Corp., Armonk, NY, USA). Continuous variables were tested for normality using the Shapiro–Wilk test and are presented as mean ± standard deviation (SD) or median with interquartile range (IQR), as appropriate. Categorical variables are presented as absolute numbers and percentages.

For group comparisons between patients with T1–3 and T4 tumors, continuous variables were analyzed using the independent-samples *t*-test or the Mann–Whitney U test, depending on data distribution. Categorical variables were compared using the chi-square test or Fisher’s exact test, as appropriate.

To evaluate the association between preoperative muscular status and locally advanced tumor stage, binary logistic regression analysis was performed with pathological T4 disease as the dependent variable. In the regression analysis, SMI was modeled as a continuous variable expressed per 5 cm^2^/m^2^ increment (SMI5), rather than per 1 cm^2^/m^2^, to yield a more clinically interpretable odds ratio. As a secondary exploratory analysis, CT-defined low SMI was also evaluated as a categorical variable. Group differences were assessed using the chi-square test, and univariable and age- and sex-adjusted logistic regression models were used to examine their association with pathological T4 disease.

Given the moderate sample size and the risk of model overfitting, the primary adjusted model was restricted to age and sex as baseline covariates. To address potential confounding by additional clinico-pathological variables, several exploratory sensitivity models were also constructed. These included separate additional adjustments for BMI, tumor localization, and emergency presentation. Finally, an exploratory fully adjusted model including SMI5, age, sex, BMI, tumor localization, metastatic status, and emergency presentation was performed. Because of the number of covariates relative to the sample size and the presence of sparse categories, this fully adjusted model was interpreted cautiously.

Emergency presentation was not included in the primary model because it may represent a clinical manifestation of locally advanced colorectal cancer rather than a simple baseline confounder. Because this was an exploratory study, no formal adjustment for multiple comparisons was applied, and secondary and sensitivity analyses were interpreted cautiously. Results are reported as odds ratios (ORs) with 95% confidence intervals (CIs). All tests were two-sided, and a *p* value of <0.05 was considered statistically significant. Missing data were minimal and were handled by complete-case analysis on a variable-by-variable basis; no imputation was performed.

### 2.5. Sensitivity Analyses

To explore the robustness of the primary results, additional exploratory sensitivity analyses were performed and interpreted cautiously. First, the full-cohort model was additionally adjusted for tumor localization (right colon, left colon, rectum). Second, a separate full-cohort model additionally adjusted for emergency presentation, given that emergency admission may be closely related to locally advanced tumor extent and acute clinical severity. Third, the regression model was repeated in patients with non-metastatic disease (M0) to reduce the potential influence of metastatic burden. Fourth, the model was repeated in elective cases only to minimize the influence of acute presentation. In all models, the association between preoperative SMI5 and pathological T4 disease was evaluated using binary logistic regression.

## 3. Results

### 3.1. General Characteristics of the Cohort

A total of 147 patients undergoing colorectal cancer surgery were included. The mean age was 69.5 ± 10.6 years, and 84 patients (57.1%) were male. Emergency presentation was recorded in 84 cases (57.1%). Tumor location was in the right colon in 61 patients (41.5%), left colon in 67 (45.6%) and rectum in 19 (12.9%). 83 (56.5%) primary anastomosis was performed and 67 of 146 evaluable patients (45.9%) had a stoma. Mean BMI was 26.7 ± 5.4 kg/m^2^, and mean SMI was 40.7 ± 10.4 cm^2^/m^2^. Baseline characteristics according to T4 status are summarized in Table 1.

**Figure 3:**
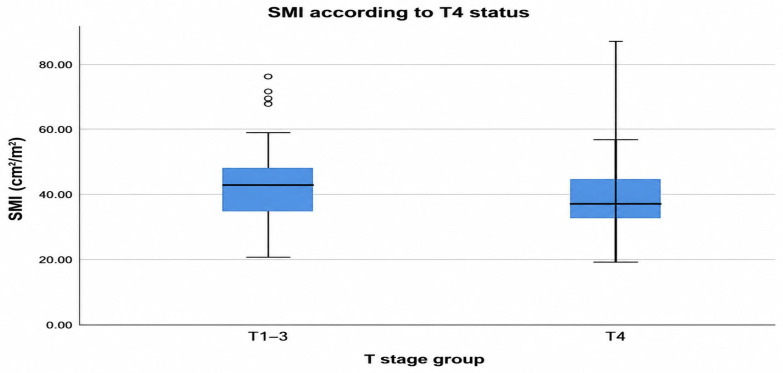
Boxplot of skeletal muscle index (SMI) according to T4 status. Median SMI was lower in patients with T4 disease than in those with T1–3 tumors (Mann–Whitney U test, *p* = 0.016). The box represents the interquartile range, the horizontal line within the box represents the median, whiskers indicate the smallest and largest non-outlier values, and dots represent outliers. Grey horizontal lines indicate the y-axis gridlines.

Patients with T4 tumors had a significantly lower SMI compared with those with T1–3 tumors (37.22 [32.61–44.58] vs. 42.85 [34.85–47.78] cm^2^/m^2^, *p* = 0.016). Emergency presentation was also significantly more frequent in the T4 group (69.3% vs. 44.4%, *p* = 0.002). Tumor localization differed significantly between groups (*p* = 0.034). No significant differences were observed for age (*p* = 0.395), metastatic disease (*p* = 0.079), nodal positivity (*p* = 0.058), or muscle attenuation (*p* = 0.892). Sex distribution and BMI showed borderline differences between groups (both *p* = 0.051), with a lower proportion of male patients and numerically lower BMI in the T4 group. The distribution of SMI according to T4 status is illustrated in Figure 3.

### 3.2. Univariable and Multivariable Analyses for T4 Stage

In univariable logistic regression, higher SMI was significantly associated with lower odds of pathological T4 disease. For each 5 cm^2^/m^2^ increase in SMI, the odds of T4 decreased by approximately 21% (OR 0.79, 95% CI 0.66–0.94, *p* = 0.008). In the primary adjusted model including age and sex, higher SMI remained significantly associated with lower odds of T4 disease (OR 0.77, 95% CI 0.63–0.94, *p* = 0.009). Age and sex were not significantly associated with T4 stage in the adjusted model (Table 2).

As a secondary categorical analysis, CT-defined low SMI was evaluated using established sex- and BMI-specific cutoffs. Low SMI was more frequent among patients with pathological T4 disease than among those with T1–3 tumors, although the unadjusted group comparison did not reach conventional statistical significance (62/75 [82.7%] vs. 50/72 [69.4%], *p* = 0.060). In logistic regression, CT-defined low SMI showed a borderline association with pathological T4 disease in univariable analysis (OR 2.10, 95% CI 0.96–4.58, *p* = 0.063) and was significantly associated with T4 disease after adjustment for age and sex (OR 2.60, 95% CI 1.12–6.00, *p* = 0.025). These findings were directionally consistent with the primary continuous SMI5 analysis, but were interpreted as exploratory because dichotomizing a continuous variable may reduce statistical power and because CT-defined low muscle mass does not represent formal sarcopenia.

### 3.3. Sensitivity Analyses

Sensitivity analyses are summarized in Table 3. The association between higher SMI5 and lower odds of pathological T4 disease remained significant after additional adjustment for BMI (OR 0.80, 95% CI 0.64–0.99, *p* = 0.039). In this model, BMI itself was not independently associated with pathological T4 disease (OR 0.97, 95% CI 0.91–1.04, *p* = 0.421). The association between SMI5 and T4 disease also remained significant after adjustment for tumor localization (OR 0.79, 95% CI 0.64–0.97, *p* = 0.024). When emergency presentation was added to the model, the association was attenuated to borderline significance (OR 0.82, 95% CI 0.66–1.00, *p* = 0.054), while emergency presentation remained independently associated with pathological T4 disease. This model was interpreted as an exploratory sensitivity analysis because emergency presentation may overlap clinically with locally advanced disease.

In the exploratory fully adjusted model including age, sex, BMI, tumor localization, metastatic status, and emergency presentation, the association between SMI5 and pathological T4 disease was further attenuated and was no longer statistically significant (OR 0.85, 95% CI 0.67–1.08, *p* = 0.195). Given the moderate sample size and the number of covariates included, this fully adjusted model was interpreted as exploratory.

In the non-metastatic subgroup (M0, *n* = 134), higher SMI5 remained significantly associated with lower odds of pathological T4 disease after adjustment for age and sex (OR 0.79, 95% CI 0.64–0.98, *p* = 0.029). In the elective subgroup (*n* = 63), the inverse association appeared stronger (OR 0.60, 95% CI 0.42–0.87, *p* = 0.007). Overall, these findings suggest that the inverse association between preoperative SMI and pathological T4 disease is directionally consistent across several exploratory analyses but may be attenuated when emergency presentation and additional clinicopathological variables are incorporated into the model.

## 4. Discussion

In this retrospective single-center cohort, patients with pathological T4 colorectal cancer had significantly lower preoperative SMI than those with T1–3 disease. Higher SMI was associated with lower odds of T4 stage in univariable analysis, and this inverse association remained significant in the primary adjusted model including age and sex. In exploratory sensitivity analyses, the association also remained significant after additional adjustment for tumor localization and in the elective-only and non-metastatic subgroups, but attenuated after additional adjustment for emergency presentation. Together, these findings suggest that reduced skeletal muscle mass may be linked to more advanced local tumor extent in colorectal cancer.

The contemporary colorectal cancer literature has mainly examined muscle depletion in relation to clinical outcomes such as postoperative morbidity, mortality, and survival, rather than in relation to the local pathological extent of the primary tumor. This trend is reflected in several individual studies and meta-analyses, showing that sarcopenia in colorectal cancer is consistently associated with increased postoperative morbidity, higher mortality, and worse long-term oncologic outcomes. Consequently, most contemporary studies have evaluated muscle depletion primarily in relation to prognosis rather than to the local pathological advancement of the primary tumor [7,10,25,29,30,31,32].

Studies incorporating pathological or clinico-pathological variables have yielded heterogeneous results. In our cohort, patients with pathological T4 tumors had lower median SMI than those with T1–3 disease (37.23 vs. 42.86 cm^2^/m^2^, *p* = 0.016), whereas muscle density did not differ significantly (28.64 vs. 28.43 HU, *p* = 0.892). Higher SMI was associated with lower odds of T4 in univariable analysis (OR per 5 cm^2^/m^2^ 0.79, 95% CI 0.66–0.94; *p* = 0.008), and this inverse association appeared even stronger in the elective subgroup (OR 0.60, 95% CI 0.42–0.87; *p* = 0.007). The report by Golder et al. is the most supportive of this pattern: in 1146 patients with colon cancer, the prevalence of low SMI increased across TNM stages I to IV, while low SMD was not significantly associated with stage, which is directionally consistent with our finding that reduced muscle quantity, but not muscle density, was linked to more advanced disease [33]. McGovern et al. provide partial support, showing that one CT-sarcopenia score based on combined low SMI and SMD was associated with TNM stage on univariable analysis, whereas another threshold definition was not, leading the authors to conclude that the relationship with stage was inconsistent and threshold-dependent [34]. By contrast, McSorley et al. reported in 322 patients with primary operable colorectal cancer that TNM stage was not significantly associated with any body composition measure [19]. Martin et al. also did not find significant differences in T or nodal status between sarcopenic and non-sarcopenic rectal cancer patients, although sarcopenic patients had more R1/R2 resections (20% vs. 8%, *p* = 0.025), more distant recurrence (26% vs. 8%, *p* = 0.002), and shorter disease-free survival [30]. Overall, the available evidence suggests that the association between muscle depletion and pathological tumor advancement is plausible but not uniform and may depend on cohort composition, stage definition, emergency case mix, and the specific sarcopenia metric applied.

A biologically plausible interpretation is that pathological T4 disease and reduced SMI may share overlapping inflammatory, nutritional, and metabolic pathways. Locally advanced tumors may be accompanied by systemic inflammation, impaired nutritional intake, and catabolic changes, all of which may contribute to reduced skeletal muscle mass [13,14,18,35,36]. However, the direction of this relationship cannot be established in the present retrospective observational study. Lower SMI may also reflect pre-existing frailty, impaired nutritional reserve, or host vulnerability rather than being a direct consequence of locally advanced disease.

Because skeletal muscle represents an important metabolic and protein reserve, reduced SMI may still be clinically relevant as a marker of diminished host reserve in patients with colorectal cancer [15,16,17]. In this context, the observed association between lower SMI and pathological T4 disease may reflect an overlap between tumor-related disease burden and host nutritional-metabolic vulnerability. Nevertheless, this interpretation should remain cautious. The present findings should not be interpreted as evidence that pathological T4 disease causes skeletal muscle depletion, but rather as a hypothesis-generating association that may involve reverse causality, shared inflammatory–nutritional mechanisms, frailty, and residual confounding.

Muscle attenuation, assessed in our study as CT radiodensity, was not significantly associated with T4 status. This may partly reflect the relatively small and heterogeneous nature of our cohort, but it also highlights that different body-composition parameters may capture different biological dimensions of muscle depletion. Importantly, most published studies discuss sarcopenia as a broader syndrome rather than isolated muscle quantity alone. Sarcopenia is not defined exclusively by low muscle mass, but also encompasses impaired muscle strength and physical performance [9]. Accordingly, CT-derived SMI does not capture the entire sarcopenia phenotype, although it remains an objective and clinically relevant marker of muscular depletion. Therefore, the present findings should be interpreted as relating to CT-derived muscle depletion rather than formally diagnosed sarcopenia. The additional categorical analysis using established CT-based cutoffs was directionally consistent with the continuous SMI analysis, although it should be interpreted cautiously because threshold-based definitions may reduce statistical power and may not fully account for population-specific variation. Although systemic inflammation likely contributes to both tumor progression and muscle wasting, it was not the primary focus of the present analysis, as our objective was to examine whether a relatively stable morphologic marker of muscular depletion was associated with local pathological tumor extent [36].

Emergency presentation was closely associated with pathological T4 disease, and inclusion of this variable attenuated the association between SMI and T4 status. This suggests that part of the observed relationship may overlap with acute clinical presentation. However, emergency presentation should not be interpreted only as a simple baseline confounder, because obstruction, perforation, or acute clinical deterioration may themselves occur as clinical manifestations of locally advanced tumors. In emergency cases, factors such as obstruction, perforation, dehydration, and acute inflammatory stress may overlap with the chronic host-related processes reflected by preoperative muscle depletion [37,38]. This may explain why the inverse SMI–T4 association appeared more pronounced in the elective subgroup, where the impact of acute presentation was likely reduced. Therefore, models with and without emergency presentation answer slightly different questions, and the attenuation observed after emergency adjustment supports a cautious interpretation of the results.

Future research should focus on clarifying whether low preoperative SMI is primarily a consequence of advanced disease and prolonged exposure to cancer-related metabolic dysregulation, or whether it reflects a pre-existing vulnerability that may facilitate faster disease progression. Multicenter prospective studies with larger cohorts and longitudinal assessments of muscle depletion would be valuable, ideally including repeated evaluation on staging or follow-up CT scans. It would also be important to determine whether combining SMI with inflammatory markers improves the prediction of advanced local disease and clinical outcomes, given the established prognostic relevance of both domains. If validated in larger cohorts, low preoperative SMI may serve as an accessible morphologic marker of reduced nutritional-metabolic reserve, advanced local disease burden and heightened host catabolism [25,39]. Because SMI can be readily obtained from routine staging CT, it may contribute to risk stratification and support early nutritional and prehabilitation strategies in elective colorectal cancer care. Although opportunities for preoperative optimization are limited in emergency settings, early recognition of high-risk patients remains clinically important [40].

Several limitations should be acknowledged. First, the retrospective single-center design limits causal inference and carries a risk of selection bias and residual confounding. Second, the moderate sample size and limited event count favored a restricted primary adjusted model. Accordingly, the main model included only age and sex as baseline covariates, whereas tumor localization and emergency presentation were examined in exploratory sensitivity analyses rather than in the primary model. Third, the cohort was heterogeneous, including both elective and emergency presentations, which may have influenced the observed associations. Fourth, body composition assessment was limited to CT-derived morphologic markers, and muscle strength or physical performance were not available; therefore, the study assessed muscle depletion rather than the full sarcopenia concept. Fifth, no longitudinal body composition data were available, so it could not be determined whether low SMI preceded tumor progression or developed as a consequence of advanced disease. Finally, CT scans were obtained from multiple institutions using different scanners, and formal intraobserver/interobserver reproducibility testing of manual segmentation was not performed. Although all segmentations were performed using a predefined attenuation threshold and manual anatomical correction under radiologist supervision, some degree of operator-dependent measurement variability cannot be excluded. Future studies should include formal reproducibility assessment, ideally using repeated segmentations and intraclass correlation coefficients. Detailed neoadjuvant treatment information was also not consistently available, and the relatively small rectal cancer subgroup limited site-specific analyses. Accordingly, these findings should be regarded as hypothesis-generating and require validation in larger prospective studies.

## 5. Conclusions

In conclusion, patients with pathological T4 colorectal cancer had lower preoperative skeletal muscle index in this cohort. This finding suggests a possible link between reduced muscle mass and more locally advanced colorectal cancer. However, because this was a retrospective observational study, we cannot determine whether advanced tumor stage contributed to muscle loss or whether low muscle mass reflected a more vulnerable patient profile. As an objective parameter readily obtainable from routine staging CT, SMI may provide additional information about patient reserve and could support risk stratification in future studies. Larger prospective studies are needed to confirm these findings and clarify their clinical relevance.

## Figures and Tables

**Table 1:** Baseline characteristics according to T4 status.

Variable	T1–3 (*n* = 72)	T4 (*n* = 75)	*p* Value
Age, years	71 (67–77)	70 (63–76)	0.395
Sex, male, *n* (%)	47 (65.3)	37 (49.3)	0.051
Emergency presentation, *n* (%)	32 (44.4)	52 (69.3)	0.002
Tumor localization, *n* (%)			0.034
Right colon	31 (43.1)	30 (40.0)	
Left colon	27 (37.5)	40 (53.3)	
Rectum	14 (19.4)	5 (6.7)	
Metastatic disease (M1), *n* (%)	3 (4.2)	10 (13.3)	0.079
Node positive disease (N+), *n* (%)	32 (45.1)	45 (60.8)	0.058
SMI cm^2^/m^2^	42.85 (34.85–47.78)	37.22 (32.61–44.58)	0.016
Muscle attenuation, HU	28.43 (23.82–33.56)	28.64 (21.78–35.65)	0.892
BMI, kg/m^2^	26.98 (23.57–30.34)	25.30 (23.11–28.19)	0.051
CT-defined low SMI, *n* (%)	50 (69.4)	62 (82.7)	0.060

Continuous variables are presented as median (IQR) and were compared using the Mann–Whitney U test. Categorical variables are presented as *n* (%) and were compared using the chi-square test or Fisher’s exact test, as appropriate.

**Table 2:** Univariable and multivariable logistic regression analyses for pathological T4 disease.

Variable	Univariate OR (95% CI)	*p* Value	Primary Adjusted OR (95% CI)	*p* Value
SMI5	0.790 (0.664–0.940)	0.008	0.767 (0.628–0.937)	0.009
AGE	0.990 (0.960–1.021)	0.525	0.969 (0.936–1.004)	0.080
MALE SEX (vs. female)	0.518 (0.267–1.006)	0.052	0.677 (0.329–1.393)	0.290

OR, odds ratio; CI, confidence interval. SMI5 represents skeletal muscle index scaled per 5 cm^2^/m^2^ increase. Primary adjusted model included SMI5, age, and sex.

**Table 3:** Exploratory sensitivity analyses for pathological T4 disease.

Model	N	Adjustment Variables	SMI5 OR (95% CI)	*p* Value
Primary adjusted model	147	Age, sex	0.767 (0.628–0.937)	0.009
BMI—adjusted model	147	Age, sex, BMI	0.796 (0.640–0.990)	0.039
Tumor localization—adjusted model	147	Age, sex, tumor localization	0.788 (0.641–0.969)	0.024
Emergency—adjusted model	147	Age, sex, emergency presentation	0.816 (0.663–1.003)	0.054
Fully adjusted exploratory model	147	Age, sex, BMI, tumor localization, M status, emergency presentation	0.854 (0.669–1.089)	0.195
Non-metastatic cohort	134	Age, sex	0.793 (0.644–0.977)	0.029
Elective cohort only	63	Age, sex	0.601 (0.416–0.869)	0.007

OR, odds ratio; CI, confidence interval; SMI5, skeletal muscle index scaled per 5 cm^2^/m^2^ increase. The table presents the effect estimate for SMI5 across exploratory sensitivity models. The fully adjusted model was interpreted cautiously because of the moderate sample size and the number of covariates included.

## Data Availability

The data is available on request because of ethical and privacy considerations of our institute.

## Abbreviations

The following abbreviations are used in this manuscript:

SMI	Skeletal muscle index
CT	Computed tomography
OR	Odds ratio
CRC	Colorectal cancer
L3	Third lumbar vertebra
CI	Confidence interval
HU	Hounsfield unit
SMA	Skeletal muscle area
SD	Standard deviation
IQR	Interquartile range
BMI	Body mass index
SMD	Skeletal muscle density
